# Uterine Rupture in Pregnancy following Two Abdominal Myomectomies and IVF

**DOI:** 10.1155/2022/6788992

**Published:** 2022-07-14

**Authors:** Marco D'Asta, Ferdinando Antonio Gulino, Carla Ettore, Valentina Dilisi, Elisa Pappalardo, Giuseppe Ettore

**Affiliations:** Department of Obstetrics and Gynaecology, Azienda di Rilievo Nazionale e di Alta Specializzazione (ARNAS) Garibaldi Nesima, Catania, Italy

## Abstract

**Objective:**

Uterine rupture (UR) during pregnancy is an obstetric emergency that could determine poor maternal and neonatal outcomes. There are many factors that could increase the risk of UR, such as a previous myomectomy. The aim of this study is to evaluate the role of a previous myomectomy in a spontaneous UR in pregnancy.

**Methods:**

A 33-year-old primigravida comes to our obstetric emergency room for pelvic pain at 29 weeks of gestation. In her medical history, there were two previous surgical operations of abdominal myomectomy, one in 2015 and one in January 2021 (6 months before conception). After 34 minutes, a pubo-subumbilical longitudinal laparotomy was performed for pathological decelerations in the cardiotocography. In the peritoneal cavity, there was 500 mL of blood serum liquid. The right arm and shoulder of the fetus were extending out of the uterus across a breach of 5 cm near the right tubal corner. A corporal incision was performed, and a healthy baby was born and moved to neonatal intensive unit care.

**Results:**

A UR can occur at any stage of pregnancy, mostly during the third trimester of pregnancy. Risk factors that increase the incidence of a uterine rupture after myomectomy include a short period (i.e., <12 months) between the myomectomy and conception, the opening of the endometrial cavity, and large myomas (with a maximum diameter above 4 cm). Uterine rupture during pregnancy after abdominal myomectomy seems to be less frequent than after a laparoscopic one.

**Conclusion:**

Uterine rupture is an obstetric emergency; it is mandatory to consider this eventuality in pregnancy, particularly in the third trimester, if there was a previous laparoscopic myomectomy in the anamnesis of the patient.

## 1. Introduction

Uterine rupture (UR) during pregnancy is an obstetric emergency that could determine poor maternal and neonatal outcomes. It can be defined as a full-thickness separation of the wall of the uterus and the covering visceral peritoneum [1].

The frequency of pregnancies after a myomectomy is growing; therefore, the cases of UR are increasing in the last few years. The incidence is approximately 1% of affected women [2].

There are many factors that could increase the risk of UR [3], such as congenital uterine anomalies, cesarean section, myomectomy, septum resection [4], cornuostomy for cornual ectopic pregnancy [5], resection of posterior deep infiltrating endometriosis [6], bilateral salpingectomy [7], and tubal reimplantation [8].

## 2. Case Report

A 33-year-old primigravida comes to our obstetric emergency room for pelvic pain at 29 weeks of gestation. After a history of infertility, the pregnancy was obtained by an in vitro fertilization technique (ICSI, intracytoplasmic sperm injection); it was a dichorionic twin pregnancy until 11° weeks of gestation, and then there was a miscarriage of one of the two embryos.

In her medical history, there were two previous surgical operations of abdominal myomectomy, one in 2015 and one in January 2021 (6 months before conception). Two myomas were described: an intramural one of the posterior uterine wall (measuring 6.0x4.5 cm) and a subserosal one localized on the uterine fundus (maximum diameter of 3 cm). In May 2021, she underwent laparoscopy for adhesiolysis.

**Figure 1:**
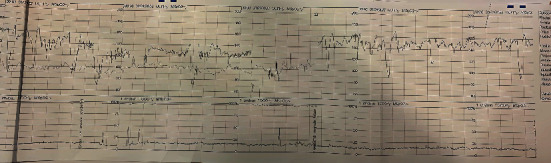
Cardiotocography at admission.

At the admission, she presented with good vital signs (blood pressure: 115/75 mmHg; heart rate: 85 bpm; arterial blood oxygen saturation: 99%; body temperature: 36.4°C). Ultrasound examination revealed a fetus in breech presentation, alive, with central placenta previa, anhydramnios, and free fluid in the pouch of Douglas. Respiratory distress syndrome prophylaxis (12 mg of betamethasone) was administered. The complete blood count results showed mild leukocytosis (WBC 16.7 10^∗^3/*μ*L; RBC 3.82 10^∗^6/*μ*L; HGB 11.3 g/dL; PLT 260 10^∗^3/*μ*L). In the cardiotocography, variable pathological decelerations were observed (Figure 1); therefore, the patient was immediately transferred to the delivery ward.

**Figure 2:**
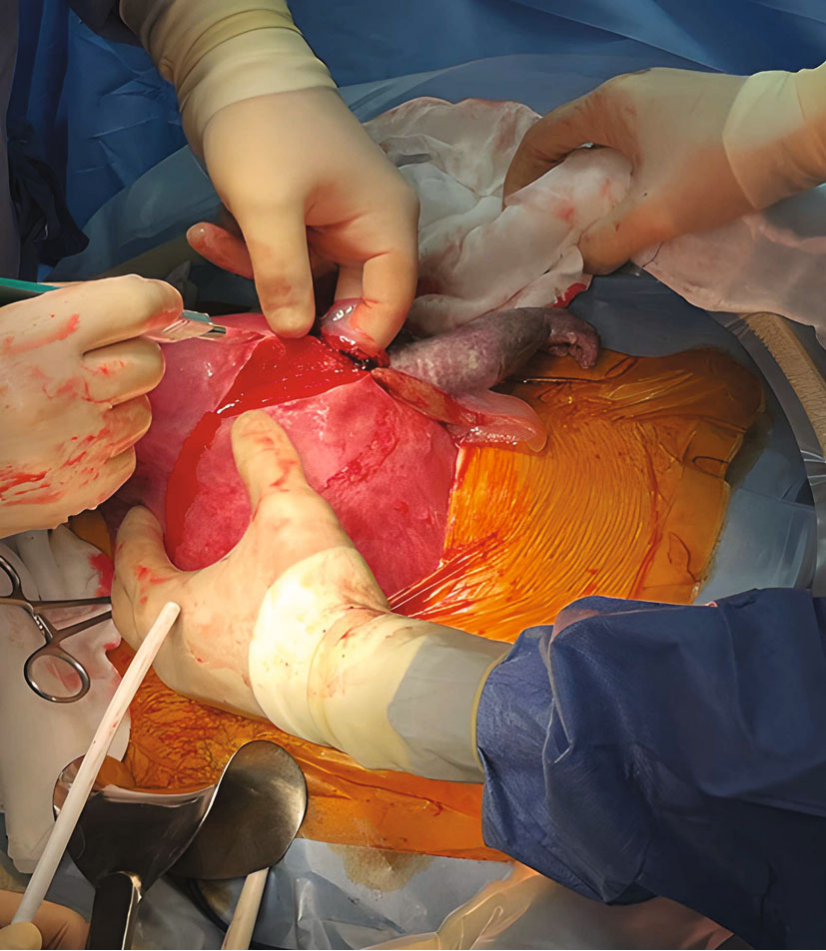
Corporal incision. Right arm of fetus falls out of breach.

After 34 minutes ,a pubo-subumbilical longitudinal laparotomy was performed. In peritoneal cavity, there was 500 mL of blood serum liquid. The right arm and shoulder of fetus were extending out of the uterus across a breach of 5 cm near right tubal corner. A corporal incision was performed, extending the breach (Figure 2).

A boy with an Apgar score of 7/8 was born and moved to neonatal intensive unit care. The breach was then closed in multilayers with Vicryl. During the caesarian section, piperacillin/tazobactam, carbetocin, sulprostone, tranexamic acid, antithrombin III, and fibrinogen were administered. The estimated blood loss was 1000 mL. The patient was then transferred to the intensive care unit; after 2 days, she was admitted to the obstetrics/gynecology ward. Her clinical conditions were stable, and she came back to home after 5 days.

## 3. Discussion

An UR can occur at any stage of pregnancy, mostly during the third trimester of pregnancy (only few cases are described during the first trimester). It can develop in an unscarred uterus, but it is more common in a scarred one.

Risk factors that increase the incidence of an uterine rupture after myomectomy include a short period (i.e., <12 months) between the myomectomy and conception and the opening of the endometrial cavity and large myomas (with maximum diameter above 4 cm) [9]. Uterine rupture during pregnancy after abdominal myomectomy seems to be less frequent than after laparoscopic one [10].

Laparoscopic myomectomy is the gold standard treatment for women with symptomatic fibroids, because of the shorter hospitalization and quicker recovery time, less postoperative pain, and less blood loss in comparison to abdominal myomectomy [3]. However, there are some procedural factors that are associated with UR, like an excessive use of electrocautery for hemostasis and inadequate laparoscopic closure of the myometrium [11].

To reduce the risk of UR after laparoscopic myomectomy, some preventive measures are advised, such as multilayer uterine closure, minimal use of electrosurgery, avoidance of the opening the endometrial cavity, and prevention of hematoma formation [12, 13].

Current evidence highlights that after abdominal myomectomies, the scars are of similar thickness of normal myometrium, whereas after laparoscopic procedures, they are strained, more contracted, and thinner than normal myometrium [10]. Most cases of ruptures (80%) occur before labor [14].

Signs and symptoms of UR are nonspecific, and thus diagnosis is troublesome. Frequent signs and symptoms include the following: fetal heart rate abnormalities (variable decelerations, bradycardia), decreased uterine tone and disappearing uterine contraction, abdominal pain, presenting part station changing, bleeding, and shock [15]. Fetal morbidity, due to catastrophic hemorrhage and/or fetal anoxia, can develop in very short time (less than an hour from the time of diagnosis to delivery) [10].

A recent study [16] showed that previous laparoscopic and abdominal myomectomies were associated with UR in pregnancy, and hysteroscopic myomectomy was associated to it at earlier gestational ages.

Considering also the increasing number of caesarean section, it has to be considered also the caesarean scar pregnancy as an important risk factor for UR [17, 18].

## 4. Conclusion

Uterine rupture is an obstetric emergency; it is mandatory to consider this eventuality in pregnancy, particularly in the third trimester, if there was a previous laparoscopic myomectomy in the anamnesis of the patient.
